# Supplementary material for: Deep neural network models reveal interplay of peripheral coding and stimulus statistics in pitch perception
Source: Nat Commun. 2021 Dec 14;12:7278. doi: 10.1038/s41467-021-27366-6 (PMC8671597; doi:10.1038/s41467-021-27366-6)
Supplement: Supplementary file 1 — Supplementary Information [file 41467_2021_27366_MOESM1_ESM.pdf]

**SUPPLEMENTARY INFORMATION**

# Deep neural network models reveal interplay of peripheral coding and stimulus statistics in pitch perception

Mark R. Saddler, Ray Gonzalez, Josh H. McDermott

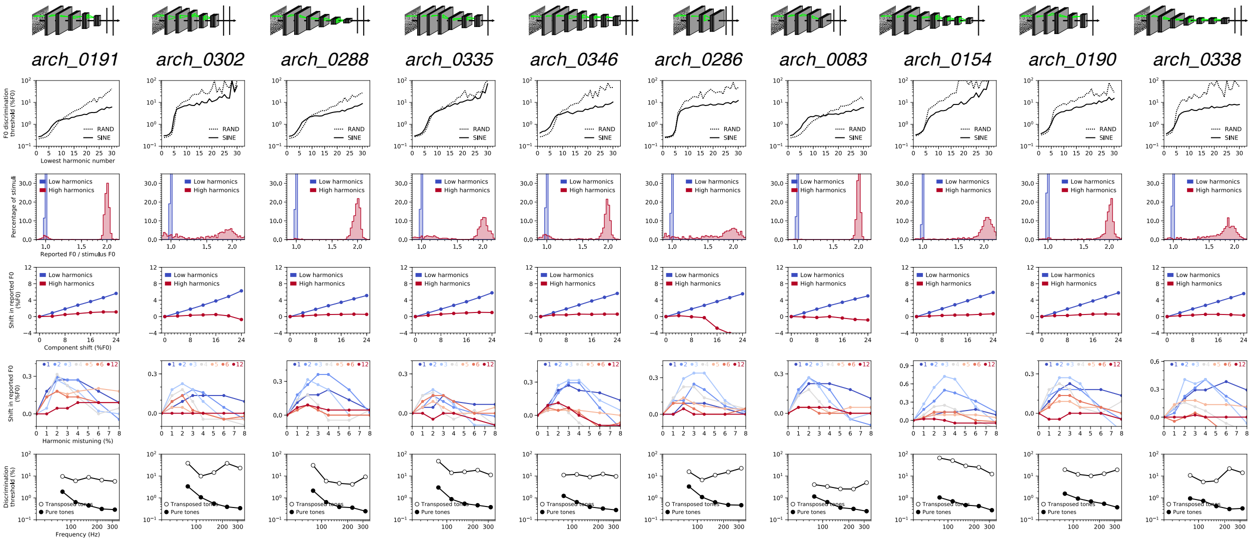


**Supplementary Figure 1.** Pitch behavior of the 10 best network architectures ranked by F0 estimation performance on natural sounds. Each column shows results from a single neural network architecture (depicted at the top). Detailed descriptions of each architecture are provided in Supplementary Table 1. The five rows in this grid correspond to the five main psychophysical experiments (see Fig. 2a-e): F0 discrimination as a function of harmonic number and phase (row 1), pitch estimation of alternating-phase stimuli (row 2), pitch estimation of frequency-shifted complexes (row 3), pitch estimation of complexes with individually mistuned harmonics (row 4), and frequency discrimination with pure and transposed tones (row 5).

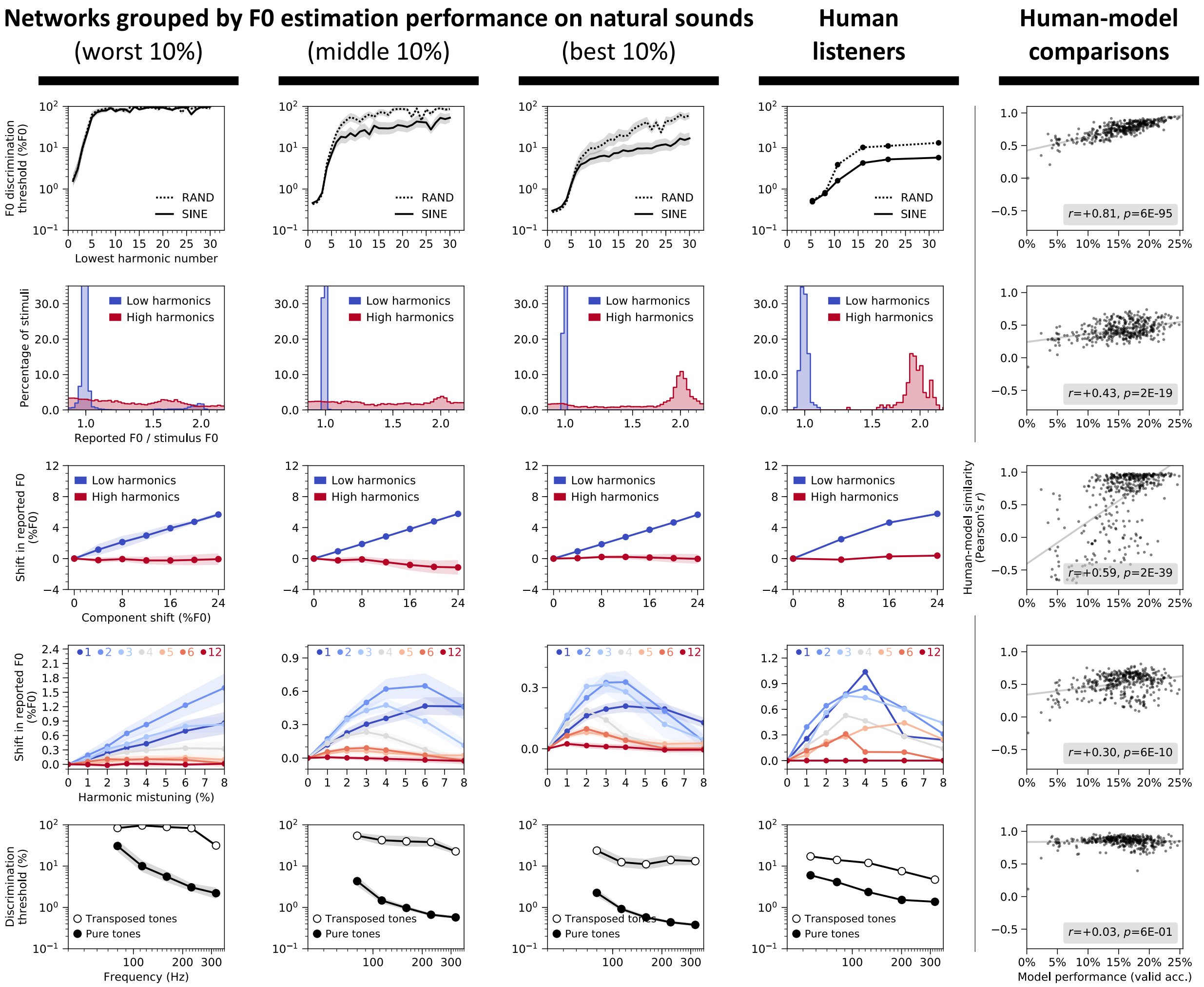


**Supplementary Figure 2.** Network architectures producing better F0 estimation for natural sounds exhibit more human-like pitch behavior. The five rows in this grid correspond to the five main psychophysical experiments (see Fig. 2a-e): F0 discrimination as a function of harmonic number and phase (row 1), pitch estimation of alternating-phase stimuli (row 2), pitch estimation of frequency-shifted complexes (row 3), pitch estimation of complexes with individually mistuned harmonics (row 4), and frequency discrimination with pure and transposed tones (row 5). First three columns show results measured from the 40 worst, middle, and best-performing network architectures (out of the 400 architectures trained in our architecture search, ranked by F0 estimation performance on natural sounds), respectively. Error bars plot bootstrapped 95% confidence intervals around the mean across the 40 networks. Human results (reproduced from Fig. 1) are shown in column 4. Column 5 contains scatter plots of human-model behavioral similarity (quantified as a correlation between the results of each model and that of humans) vs. validation set accuracy for all trained networks (reproduced from Fig. 3). Pearson correlations between validation set accuracy and human-model similarity for each experiment are noted in the legends.

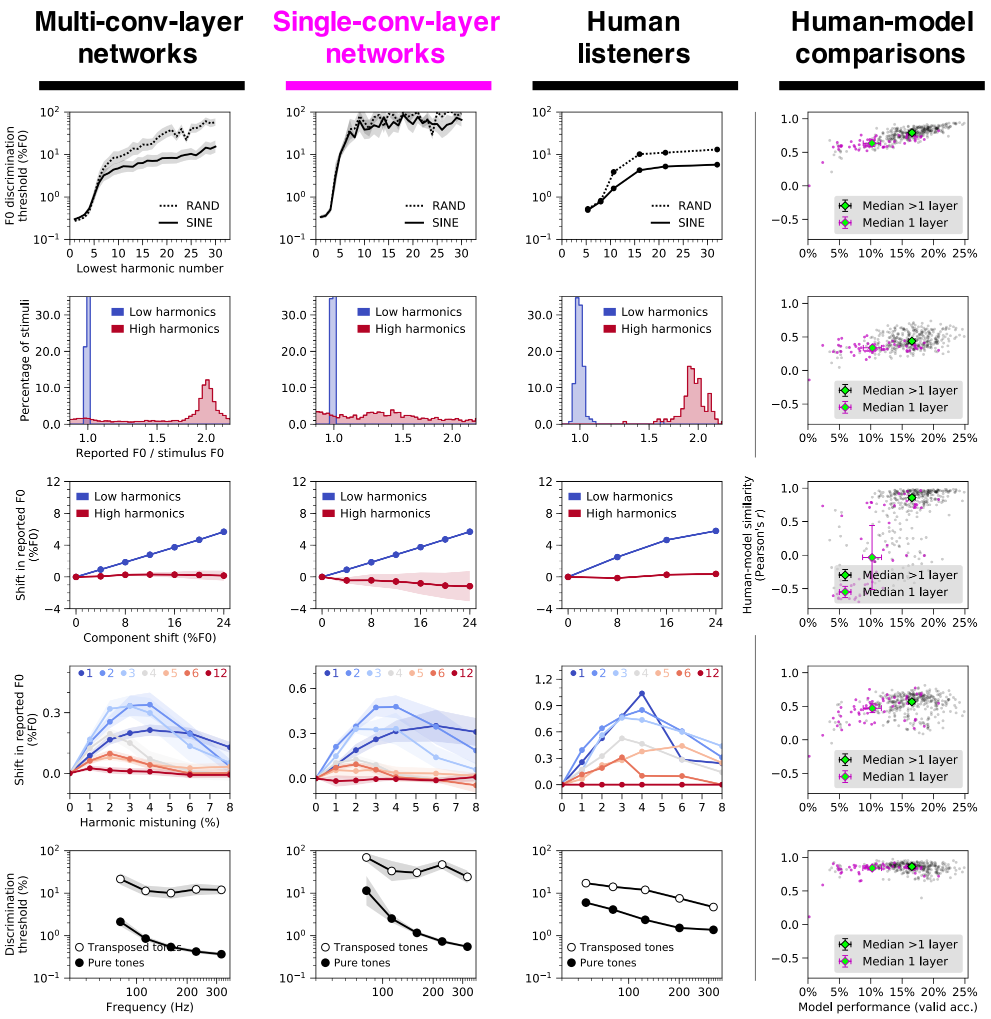


**Supplementary Figure 3.** Deep networks better account for human psychophysical behavior than networks with just one convolutional layer. Of the 400 randomly-generated networks we considered, 54 contained only one convolutional layer. These 54 single-convolutional-layer networks produced lower validation set accuracies (z=7.31, p<0.001, Wilcoxon rank-sum test), shown on the x-axis of the graphs in the right column. The single-convolutional-layer networks also produce less human-like psychophysical results. The five rows in this grid correspond to the five main psychophysical experiments (see Fig. 2a-e): F0 discrimination as a function of harmonic number and phase (row 1), pitch estimation of alternating-phase stimuli (row 2), pitch estimation of frequency-shifted complexes (row 3), pitch estimation of complexes with individually mistuned harmonics (row 4), and frequency discrimination with pure and transposed tones (row 5). In column 1, network psychophysical results are shown averaged across the top 10% (34 of 346) of networks (ranked by validation set performance) containing more than one convolutional layer. In column 2, results are shown averaged across the top 10% (5 of 54) of networks containing just one convolutional layer. Human results are shown in column 3. Column 4 contains scatter plots of human-model behavioral similarity (quantified as a correlation between the results of each model and that of humans) vs. validation set accuracy for all trained networks. Gray and magenta data points correspond to multi-convolutional-layer and single-convolutional-layer networks, respectively. Median data points are included for each group to illustrate how single-layer networks generally both performed worse on the F0 estimation task and produced poorer matches to human behavior. Error bars plot bootstrapped 95% confidence intervals around the mean across architectures. When pooled across all five experiments, human-model similarity was lower for single-convolutional-layer networks than the remaining 346 multi-convolutional-layer networks (z=9.24, p<0.001, two-sided Wilcoxon rank-sum test). Moreover, the top 40% of networks ranked according to overall human-model similarity consisted entirely of multi-convolutional-layer networks.

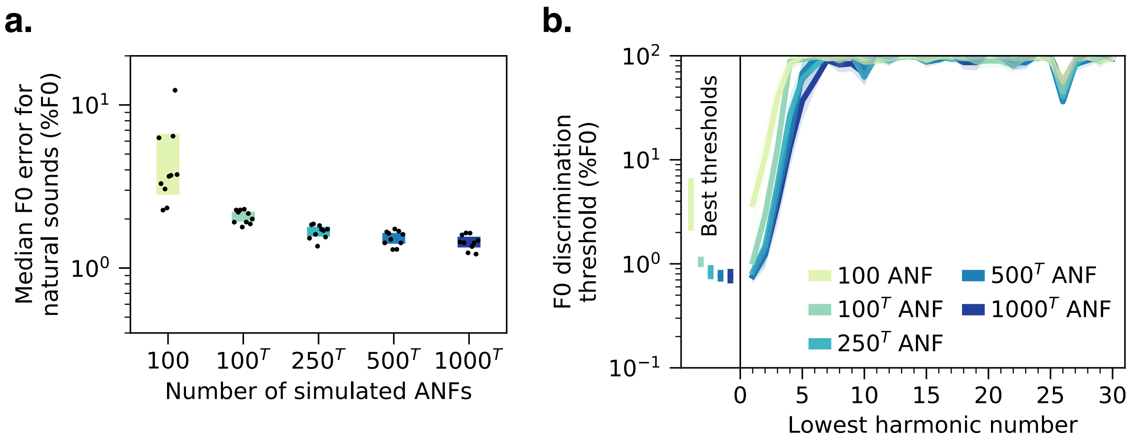


**Supplementary Figure 4.** Effect of number of auditory nerve fibers on F0 estimation error and discrimination thresholds measured from networks without spike-timing information. **(a)** Median F0 estimation error on the natural sounds validation set for 10 networks trained and tested with five different peripheral model configurations varying in the number of input auditory nerve fibers (ANFs) and in whether the frequency and time dimensions were "transposed" (indicated by superscript T). The network architectures were constrained to take a 100-by-1000 array as input. The default input representation was 100 frequency channels (auditory nerve fibers) by 1000 timesteps. A transposed input representation consists of 1000 frequency channels by 100 timesteps. To manipulate the number of nerve fibers while keeping the size of the input representation fixed, transposed peripheral representations with fewer than 1000 nerve fibers were upsampled to 1000 (via linear interpolation) along the frequency (auditory nerve fiber) dimension. Time was sampled at 2 kHz to yield 100 timesteps. For all networks included in the plot, the IHC filter cutoff frequency was set to 50 Hz to eliminate all phase-locked temporal information (see Fig. 5). **(b)** F0 discrimination thresholds as a function of lowest harmonic number of synthetic tones, measured from the same networks as **(a)**. The best thresholds are re-plotted to the left of the main axes. Error bars plot bootstrapped 95% confidence intervals around the mean across the 10 best network architectures.

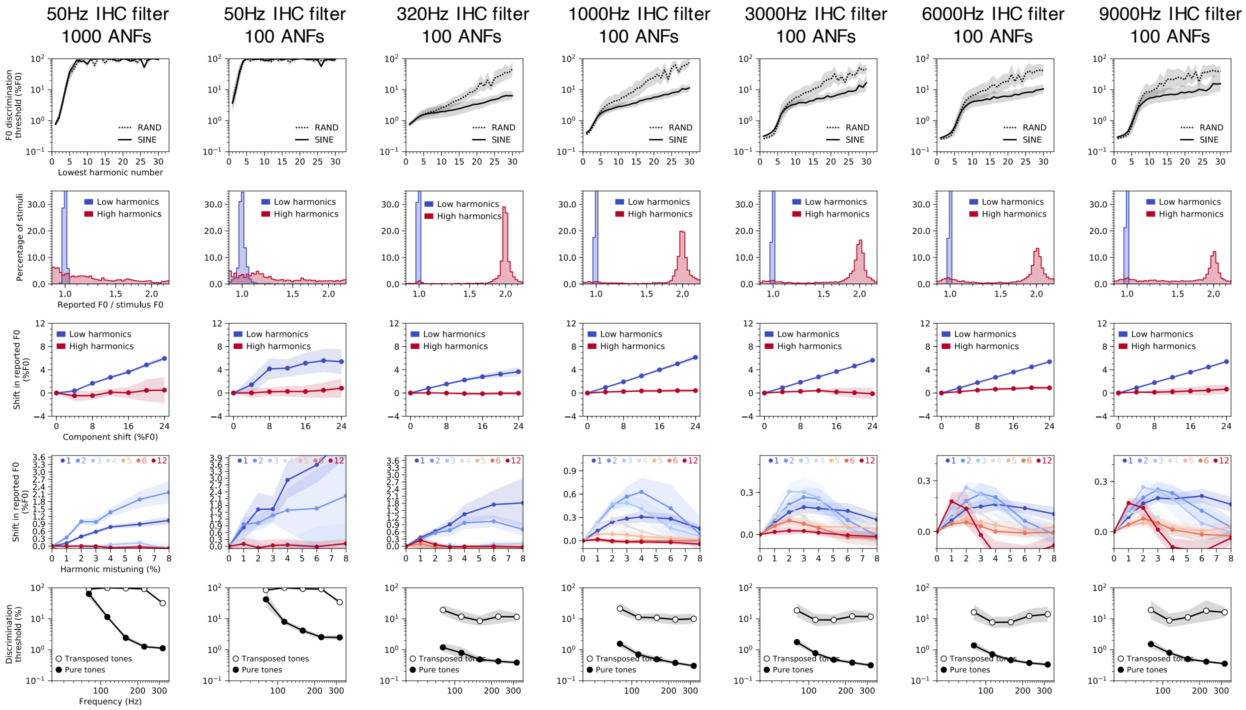


**Supplementary Figure 5.** Effects of phase locking cutoff on network pitch behavior. Columns correspond to networks trained and tested with seven different configurations of the peripheral auditory model. Configurations differed in the upper frequency limit of auditory nerve phase locking (inner hair cell lowpass filter cutoff) and the number of auditory nerve fibers (ANFs) (see Fig. 5). Rows correspond to the five main psychophysical experiments (see Fig. 2a-e): F0 discrimination as a function of harmonic number and phase (row 1), pitch estimation of alternating-phase stimuli (row 2), pitch estimation of frequency-shifted complexes (row 3), pitch estimation of complexes with individually mistuned harmonics (row 4), and frequency discrimination with pure and transposed tones (row 5). Error bars plot bootstrapped 95% confidence intervals around the mean across the 10 best network architectures.

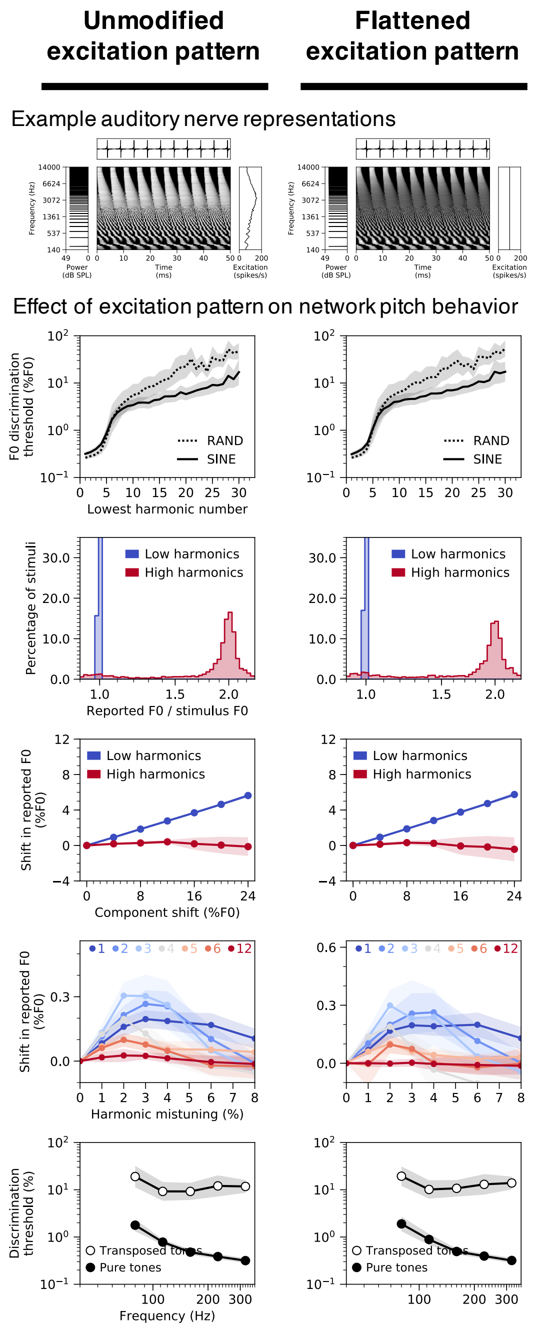


**Supplementary Figure 6.** Networks do not rely on place cues to F0 in the excitation pattern to produce human-like pitch behavior. **(a)** Simulated peripheral representations of the same stimulus (harmonic tone with 200 Hz F0) with unmodified (left column) or flattened (right column) time-averaged excitation patterns. The peaks and valleys in the unmodified excitation pattern, which provide place cues to F0, were eliminated by separately scaling each frequency channel of the nerve representation to have the same time-averaged response. **(b)** Psychophysical results from the best-performing network architecture tested on auditory nerve representations with either unmodified or flattened excitation patterns. In both cases, the model was trained on auditory nerve representations with unmodified excitation patterns. Rows correspond to the five main psychophysical experiments (see Fig. 2a-e): F0 discrimination as a function of harmonic number and phase (row 1), pitch estimation of alternating-phase stimuli (row 2), frequency-shifted complexes (row 3), and complexes with individually mistuned harmonics (row 4), and frequency discrimination with pure and transposed tones (row 5). Error bars plot bootstrapped 95% confidence intervals around the mean across the 10 best network architectures.

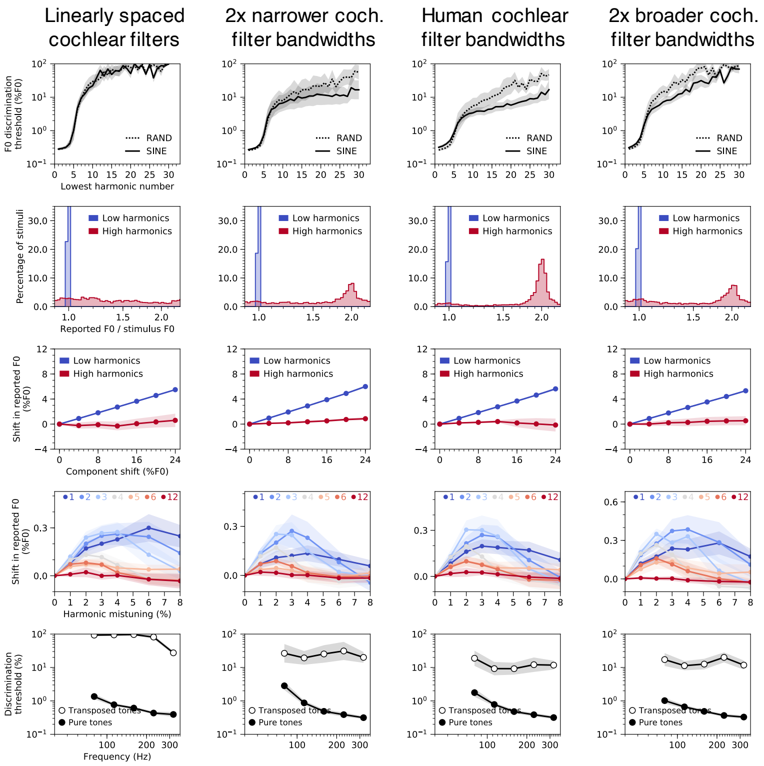


**Supplementary Figure 7.** Effects of altered cochlear frequency selectivity on network pitch behavior. The four columns correspond to networks trained and tested with four different settings of cochlear frequency selectivity: linearly spaced cochlear filters with constant bandwidths (column 1), cochlear filters with bandwidths two times narrower than those estimated for normal hearing-humans but normally spaced (i.e., evenly spaced on an ERB scale, to best approximate the spacing believed to characterize the ear) (column 2), normally spaced cochlear filters with bandwidths matched to those of normal-hearing humans (column 3), and cochlear filters with two times broader bandwidths but normally spaced (column 4) (see Fig. 6). Rows correspond to the five main psychophysical experiments (see Fig. 2a-e): F0 discrimination as a function of harmonic number and phase (row 1), pitch estimation of alternating-phase stimuli (row 2), pitch estimation of frequency-shifted complexes (row 3), pitch estimation of complexes with individually mistuned harmonics (row 4), and frequency discrimination with pure and transposed tones (row 5). Error bars plot bootstrapped 95% confidence intervals around the mean across the 10 best network architectures. Psychophysical results were qualitatively robust to changes in peripheral frequency tuning. The main exceptions were the effects of harmonic phase, which were reduced for the linearly spaced models (correlations between human and model results for the phase randomization and alternating phase experiments were lower in the linearly spaced condition than in the human tuning condition; phase randomization: $t(18)=3.13$, $p<0.01$, $d=1.40$; alternating phase: $t(18)=6.50$, $p<0.001$, $d=2.91$; two-sided two-sample t-tests). These results are to be expected because the sharp tuning of the linearly spaced filters (Fig. 6B) results in less interaction between adjacent harmonics, which is believed to drive phase effects.

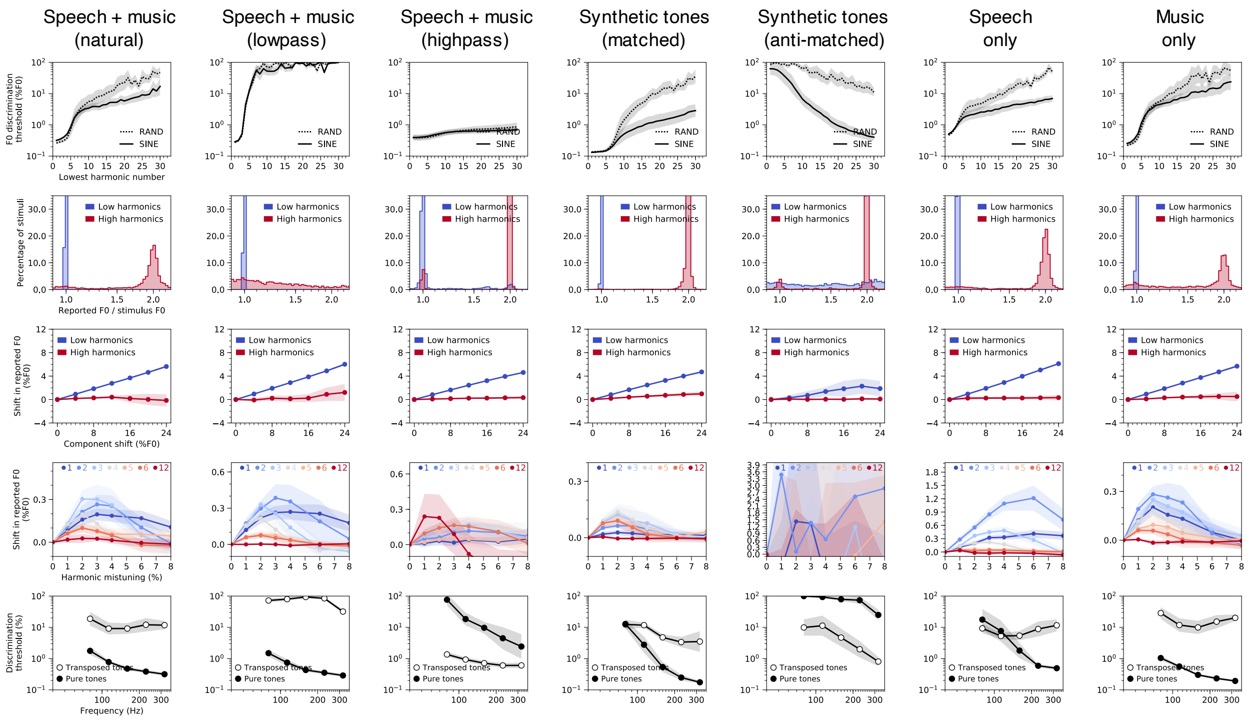


**Supplementary Figure 8.** Effects of training set sound statistics on network pitch behavior. Columns correspond to different training datasets (described in column titles). Rows correspond to the five main psychophysical experiments (see Fig. 2a-e): F0 discrimination as a function of harmonic number and phase (row 1), pitch estimation of alternating-phase stimuli (row 2), pitch estimation of frequency-shifted complexes (row 3), pitch estimation of complexes with individually mistuned harmonics (row 4), and frequency discrimination with pure and transposed tones (row 5). Results are shown for the best-performing network architecture, averaged across 10 instances of the architecture trained from different random initializations. Error bars plot bootstrapped 95% confidence intervals around the mean across the 10 best network architectures.

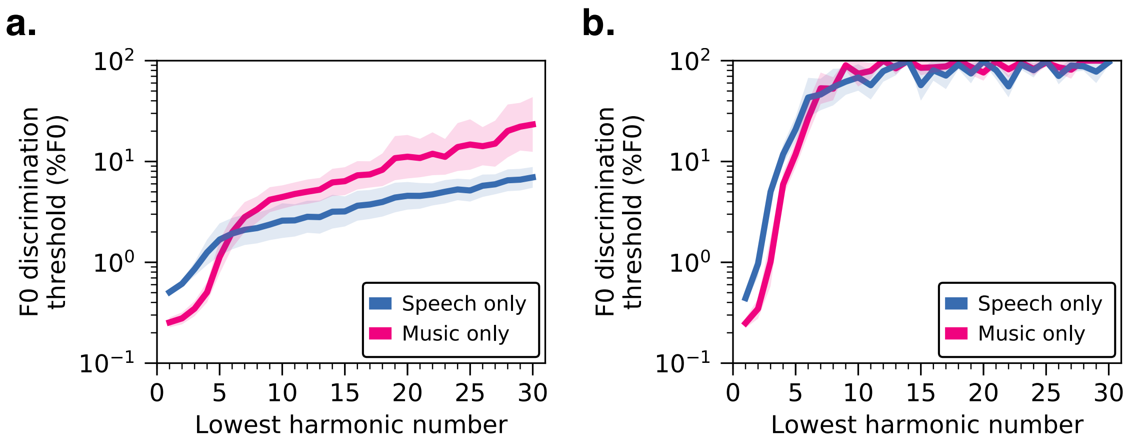


**Supplementary Figure 9.** F0 discrimination thresholds as a function of lowest harmonic number, measured from networks trained separately on speech-only and music-only datasets. **(a)** Results from networks trained on simulated auditory nerve representations produced by a fixed peripheral auditory model (reproduced from Fig. 7c). **(b)** Results from networks trained directly on sound waveforms (first-layer "cochlear" filters were learned alongside the rest of the network weights; see Fig. 4). Error bars plot bootstrapped 95% confidence intervals around the mean across the 10 best network architectures.

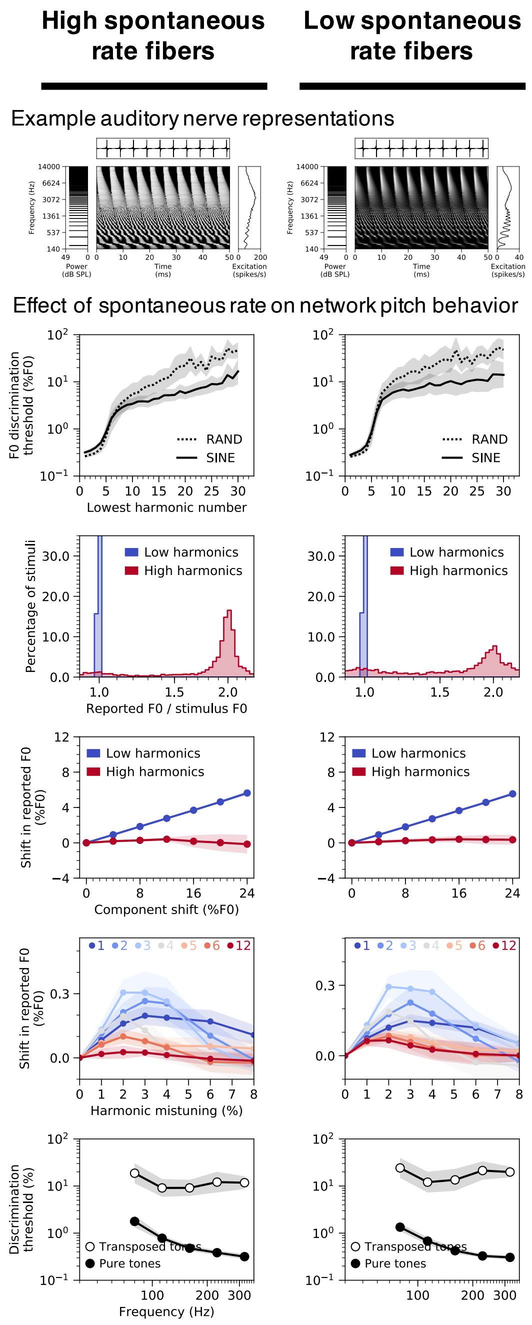


**Supplementary Figure 10.** Effects of auditory nerve fiber type (high vs. low spontaneous rate) on network pitch behavior. **(a)** Simulated peripheral representations of the same stimulus (harmonic tone with 200 Hz F0) with high (70 spikes/s; left column) and low (0.1 spikes/s; right column) spontaneous rate auditory nerve fibers. **(b)** Psychophysical results from the best-performing network architecture trained and tested with each of the two different nerve fiber types. Rows correspond to the five main psychophysical experiments (see Fig. 2a-e): F0 discrimination as a function of harmonic number and phase (row 1), pitch estimation of alternating-phase stimuli (row 2), frequency-shifted complexes (row 3), and complexes with individually mistuned harmonics (row 4), and frequency discrimination with pure and transposed tones (row 5). Error bars plot bootstrapped 95% confidence intervals around the mean across the 10 best network architectures.

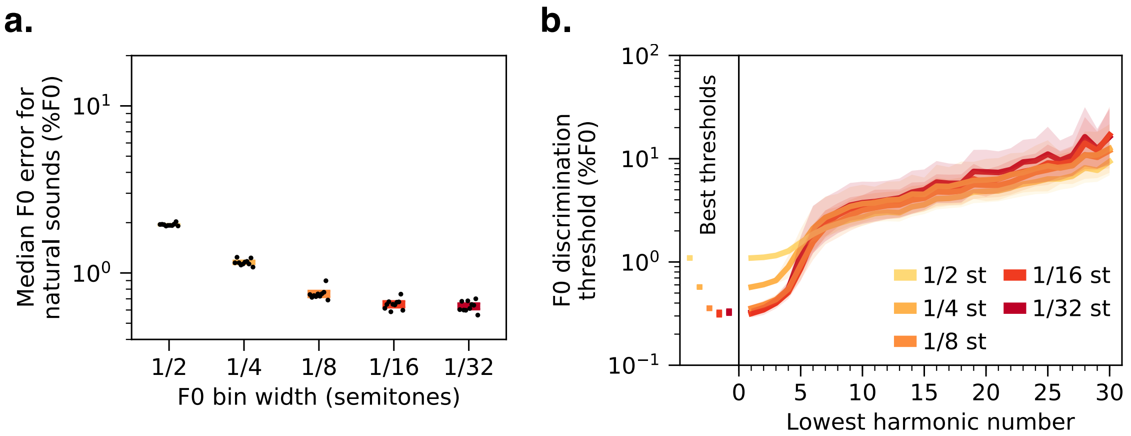


**Supplementary Figure 11.** Effects of F0 classification bin width on F0 estimation error and discrimination thresholds. **(a)** Median F0 estimation error on the natural sounds validation set for 10 networks trained and tested with five different F0 classification bin widths. **(b)** F0 discrimination thresholds as a function of lowest harmonic number of synthetic tones, measured from the same networks as **a**. The best thresholds are re-plotted to the left of the main axes. Error bars plot bootstrapped 95% confidence intervals around the mean across the 10 best network architectures.

| Architecture | *arch_0191* | *arch_0302* | *arch_0288* | *arch_0335* | *arch_0346* | *arch_0286* | *arch_0083* | *arch_0154* | *arch_0190* | *arch_0338* |
|---|---|---|---|---|---|---|---|---|---|---|
| Validation set rank (of 400) | 1 | 2 | 3 | 4 | 5 | 6 | 7 | 8 | 9 | 10 |
| Validation set accuracy (%) | 24.9 | 24.6 | 24.1 | 23.6 | 23.6 | 23.5 | 23.4 | 23.2 | 22.7 | 22.7 |
| **Operation** | input [100, 1000, 1] | input [100, 1000, 1] | input [100, 1000, 1] | input [100, 1000, 1] | input [100, 1000, 1] | input [100, 1000, 1] | input [100, 1000, 1] | input [100, 1000, 1] | input [100, 1000, 1] | input [100, 1000, 1] |
| 1 | conv_1 [2, 83, 32] | conv_1 [1, 250, 32] | conv_1 [3, 77, 64] | conv_1 [1, 71, 32] | conv_1 [3, 53, 32] | conv_1 [1, 180, 64] | conv_1 [2, 82, 32] | conv_1 [1, 70, 64] | conv_1 [2, 97, 32] | conv_1 [2, 110, 64] |
| 2 | relu_1 [99, 918, 32] | relu_1 [100, 751, 32] | relu_1 [98, 924, 64] | relu_1 [100, 930, 32] | relu_1 [98, 948, 32] | relu_1 [100, 821, 64] | relu_1 [99, 919, 32] | relu_1 [100, 931, 64] | relu_1 [99, 904, 32] | relu_1 [99, 891, 64] |
| 3 | pool_1 [1, 2] | pool_1 [1, 5] | pool_1 [1, 2] | pool_1 [1, 1] | pool_1 [1, 2] | pool_1 [2, 6] | pool_1 [1, 2] | pool_1 [1, 5] | pool_1 [1, 6] | pool_1 [3, 2] |
| 4 | norm_1 [99, 459, 32] | norm_1 [100, 151, 32] | norm_1 [98, 462, 64] | norm_1 [100, 930, 32] | norm_1 [98, 474, 32] | norm_1 [50, 137, 64] | norm_1 [99, 460, 32] | norm_1 [100, 187, 64] | norm_1 [99, 151, 32] | norm_1 [33, 446, 64] |
| 5 | conv_2 [1, 164, 64] | conv_2 [19, 11, 64] | conv_2 [1, 193, 128] | conv_2 [1, 114, 32] | conv_2 [1, 60, 64] | conv_2 [2, 37, 128] | conv_2 [1, 162, 64] | conv_2 [7, 21, 128] | conv_2 [5, 11, 64] | conv_2 [1, 126, 128] |
| 6 | relu_2 [99, 296, 64] | relu_2 [82, 141, 64] | relu_2 [98, 270, 128] | relu_2 [100, 817, 32] | relu_2 [98, 415, 64] | relu_2 [49, 101, 128] | relu_2 [99, 299, 64] | relu_2 [94, 167, 128] | relu_2 [95, 141, 64] | relu_2 [33, 321, 128] |
| 7 | pool_2 [3, 7] | pool_2 [1, 7] | pool_2 [4, 3] | pool_2 [1, 3] | pool_2 [2, 4] | pool_2 [1, 1] | pool_2 [2, 2] | pool_2 [4, 3] | pool_2 [1, 1] | pool_2 [3, 3] |
| 8 | norm_2 [33, 43, 64] | norm_2 [82, 21, 64] | norm_2 [25, 90, 128] | norm_2 [100, 273, 32] | norm_2 [49, 104, 64] | norm_2 [49, 101, 128] | norm_2 [50, 150, 64] | norm_2 [24, 56, 128] | norm_2 [95, 141, 64] | norm_2 [11, 107, 128] |
| 9 | conv_3 [5, 9, 128] | conv_3 [12, 9, 128] | conv_3 [8, 10, 128] | conv_3 [1, 86, 64] | conv_3 [3, 46, 128] | conv_3 [15, 10, 128] | conv_3 [1, 72, 128] | conv_3 [4, 26, 256] | conv_3 [1, 56, 128] | conv_3 [4, 30, 256] |
| 10 | relu_3 [29, 35, 128] | relu_3 [71, 13, 128] | relu_3 [18, 81, 128] | relu_3 [100, 188, 64] | relu_3 [47, 59, 128] | relu_3 [35, 92, 128] | relu_3 [50, 79, 128] | relu_3 [21, 31, 256] | relu_3 [95, 86, 128] | relu_3 [8, 78, 256] |
| 11 | pool_3 [1, 7] | pool_3 [3, 1] | pool_3 [2, 6] | pool_3 [4, 1] | pool_3 [1, 6] | pool_3 [1, 1] | pool_3 [4, 2] | pool_3 [1, 6] | pool_3 [4, 7] | pool_3 [2, 5] |
| 12 | norm_3 [29, 5, 128] | norm_3 [24, 13, 128] | norm_3 [9, 14, 128] | norm_3 [25, 188, 64] | norm_3 [47, 10, 128] | norm_3 [35, 92, 128] | norm_3 [13, 40, 128] | norm_3 [21, 6, 256] | norm_3 [24, 13, 128] | norm_3 [4, 16, 256] |
| 13 | conv_4 [4, 3, 256] | conv_4 [7, 7, 256] | conv_4 [2, 2, 256] | conv_4 [13, 13, 128] | conv_4 [8, 1, 256] | fc_1 [512] | conv_4 [6, 3, 128] | conv_4 [2, 1, 512] | conv_4 [8, 5, 256] | conv_4 [1, 5, 256] |
| 14 | relu_4 [26, 3, 256] | relu_4 [18, 7, 256] | relu_4 [8, 13, 256] | relu_4 [13, 176, 128] | relu_4 [40, 10, 256] | relu_fc_1 [512] | relu_4 [8, 38, 128] | relu_4 [20, 6, 512] | relu_4 [17, 9, 256] | relu_4 [4, 12, 256] |
| 15 | pool_4 [2, 1] | pool_4 [1, 1] | pool_4 [2, 2] | pool_4 [1, 8] | pool_4 [2, 2] | norm_fc_1 [512] | pool_4 [2, 5] | pool_4 [2, 1] | pool_4 [1, 2] | pool_4 [1, 3] |
| 16 | norm_4 [13, 3, 256] | norm_4 [18, 7, 256] | norm_4 [4, 7, 256] | norm_4 [13, 22, 128] | norm_4 [20, 5, 256] | dropout [512] | norm_4 [4, 8, 128] | norm_4 [10, 6, 512] | norm_4 [17, 5, 256] | norm_4 [4, 4, 256] |
| 17 | conv_5 [5, 2, 512] | conv_5 [5, 3, 512] | conv_5 [2, 1, 512] | conv_5 [2, 10, 256] | conv_5 [7, 2, 256] | fc_out [700] | fc_1 [128] | conv_5 [5, 3, 256] | conv_5 [1, 3, 256] | conv_5 [2, 2, 512] |
| 18 | relu_5 [9, 2, 512] | relu_5 [14, 5, 512] | relu_5 [3, 7, 512] | relu_5 [12, 13, 256] | relu_5 [14, 4, 256] | | relu_fc_1 [128] | relu_5 [6, 4, 256] | relu_5 [17, 3, 256] | relu_5 [3, 3, 512] |
| 19 | pool_5 [1, 1] | pool_5 [3, 1] | pool_5 [1, 1] | pool_5 [1, 3] | pool_5 [1, 1] | | norm_fc_1 [128] | pool_5 [1, 1] | pool_5 [2, 1] | pool_5 [1, 1] |
| 20 | norm_5 [9, 2, 512] | norm_5 [5, 5, 512] | norm_5 [3, 7, 256] | norm_5 [12, 5, 256] | norm_5 [14, 4, 256] | | dropout [128] | norm_5 [6, 4, 256] | norm_5 [9, 3, 256] | norm_5 [3, 3, 512] |
| 21 | fc_1 [256] | fc_1 [1024] | conv_6 [2, 4, 1024] | conv_6 [3, 2, 512] | conv_6 [2, 2, 512] | | fc_out [700] | conv_6 [2, 2, 256] | dropout [6912] | conv_6 [1, 1, 1024] |
| 22 | relu_fc_1 [256] | relu_fc_1 [1024] | relu_6 [2, 4, 1024] | relu_6 [10, 4, 512] | relu_6 [13, 3, 512] | | | relu_6 [5, 3, 256] | fc_out [700] | relu_6 [3, 3, 1024] |
| 23 | norm_fc_1 [256] | norm_fc_1 [1024] | pool_6 [1, 1] | pool_6 [2, 1] | pool_6 [2, 1] | | | pool_6 [1, 1] | | pool_6 [1, 1] |
| 24 | dropout [256] | dropout [1024] | norm_6 [2, 4, 1024] | norm_6 [5, 4, 512] | norm_6 [7, 3, 512] | | | norm_6 [5, 3, 256] | | norm_6 [3, 3, 1024] |
| 25 | fc_out [700] | fc_out [700] | fc_1 [256] | dropout [10240] | conv_7 [1, 1, 512] | | | conv_7 [3, 1, 256] | | conv_7 [1, 1, 1024] |
| 26 | | | relu_fc_1 [256] | fc_out [700] | relu_7 [7, 3, 512] | | | relu_7 [3, 3, 256] | | relu_7 [3, 3, 1024] |
| 27 | | | norm_fc_1 [256] | | pool_7 [1, 1] | | | pool_7 [1, 1] | | pool_7 [1, 1] |
| 28 | | | dropout [256] | | norm_7 [7, 3, 512] | | | norm_7 [3, 3, 256] | | norm_7 [3, 3, 1024] |
| 29 | | | fc_out [700] | | fc_1 [512] | | | dropout [2304] | | fc_1 [256] |
| 30 | | | | | relu_fc_1 [512] | | | fc_out [700] | | relu_fc_1 [256] |
| 31 | | | | | norm_fc_1 [512] | | | | | norm_fc_1 [256] |
| 32 | | | | | dropout [512] | | | | | dropout [256] |
| 33 | | | | | fc_out [700] | | | | | fc_out [700] |
| 34 | | | | | | | | | | |

**Supplementary Table 1.** Details of 10 best network architectures. Columns correspond to 10 distinct convolutional neural network architectures (the 10 best-performing networks identified in our random architecture search). Rows include descriptors of constituent network operations. Grey horizontal bands group operations by convolutional layer. With two exceptions, all results figures present results averaged across all 10 architectures. The two exceptions are Fig. 9a, which features only the best-performing architecture (arch_0191), and Supplementary Fig. 1, which displays results separately for each of these 10 architectures. Legend:

- $conv\ [h, w, k]$ : convolutional layer with $h =$ kernel height (frequency dimension), $w =$ kernel width (time dimension), and $k =$ number of kernels
- $relu\ [N_f, N_t, N_k]$ : rectified linear unit activation function operating on inputs with the specified shape ($N_f =$ frequency dimension, $N_t$ = time dimension, and $N_k$ = kernel dimension)
- $pool\ [s_f, s_t]$: weighted averaged pooling operation with stride $s_f$ in the frequency dimension and stride $s_t$ in the time dimension
- $norm\ [N_f, N_t, N_k]$ : batch normalization operating on inputs with the specified shape ($N_f =$ frequency dimension, $N_t$ = time dimension, and $N_k$ = kernel dimension)
- $fc\ [N]$ : fully-connected layer with $N$ units
- $dropout$ : dropout regularization with 50% dropout rate
